# Integrin-mediated internalization of *Staphylococcus aureus* does not require vinculin

**DOI:** 10.1186/1471-2121-14-2

**Published:** 2013-01-07

**Authors:** Marina Borisova, Yong Shi, Alexander Buntru, Susanne Wörner, Wolfgang H Ziegler, Christof R Hauck

**Affiliations:** 1 Lehrstuhl Zellbiologie, Universität Konstanz, Postfach X908, 78457, Konstanz, Germany; 2 Konstanz Research School Chemical Biology, Universität Konstanz, 78457, Konstanz, Germany; 3 Hannover Medical School, Dept. of Paediatric Kidney, Liver and Metabolic Diseases, 30625, Hannover, Germany

**Keywords:** *Staphylococcus aureus*, Bacterial adhesion, Endocytosis, Fibronectin, Host cell invasion, Integrin, Vinculin

## Abstract

**Background:**

Disease manifestations of *Staphylococcus aureus* are connected to the fibronectin (Fn)-binding capacity of these Gram-positive pathogens. Fn deposition on the surface of *S. aureus* allows engagement of α5β1 integrins and triggers uptake by host cells. For several integrin- and actin-associated cytoplasmic proteins, including FAK, Src, N-WASP, tensin and cortactin, a functional role during bacterial invasion has been demonstrated. As reorganization of the actin cytoskeleton is critical for bacterial entry, we investigated whether vinculin, an essential protein linking integrins with the actin cytoskeleton, may contribute to the integrin-mediated internalization of *S. aureus*.

**Results:**

Complementation of vinculin in vinculin -/- cells, vinculin overexpression, as well as shRNA-mediated vinculin knock-down in different eukaryotic cell types demonstrate, that vinculin does not have a functional role during the integrin-mediated uptake of *S. aureus*.

**Conclusions:**

Our results suggest that vinculin is insignificant for the integrin-mediated uptake of *S. aureus* despite the critical role of vinculin as a linker between integrins and F-actin.

## Background

*Staphylococcus aureus* is often associated with nosocomial infections, which can lead to life threatening conditions such as acute endocarditis and bacteremia. Pathogenesis by *S. aureus* involves several bacterial virulence factors including exotoxins and adhesins [1]. Six different adhesins of *S. aureus* (FnBPA, FnBPB, Eap, Emb, Ebh and Aaa/Sle1) were found to bind to the extracellular matrix (ECM) protein fibronectin [2]. In particular, bacterial cell wall anchored fibronectin binding proteins (FnBPs) can capture soluble fibronectin from host plasma [3]. Binding to fibronectin is mediated by several repeats within FnBP, which interact with the amino-terminal fibronectin type I domains of this host glycoprotein [4]. Via an RGD motif within one of the fibronectin type III domains, *S. aureus*-associated fibronectin is able to bind with high affinity to integrin α5β1. Accordingly, fibronectin can serve as a molecular bridge connecting *S. aureus* with host cell receptors [5,6]. Moreover, the FnBP-fibronectin mediated engagement of integrins triggers internalization of the microbes by non-professional phagocytes such as epithelial cells, endothelial cells, keratinocytes, and fibroblasts [7-10]. Several investigations demonstrate the importance of the FnBP-mediated invasion process *in vivo*. In a rat model of endocarditis, *S. aureus* strains with reduced fibronectin-binding capacity showed a decreased ability to colonize damaged heart valves [11]. In addition, FnBP expression enhances the capacity of *S. aureus* to colonize mammary glands and invade mammary epithelial cells in a mouse model of mastitis [12]. Exogenous expression of *S. aureus* FnBP in non-pathogenic *Lactobacillus lactis* allows these bacteria to colonize damaged heart valves and to spread to the spleen in a mouse model of endocarditis [13]. Therefore, FnBP-mediated host cell contact and cellular invasion appear to contribute to *S. aureus* survival and persistence within the infected host [14]. As FnBP-related proteins are found in other human pathogens and as integrin-mediated host cell internalization appears critical for certain manifestations of *S. aureus* infections, a better understanding of the molecular mechanisms guiding FnBP-initiated uptake is warranted.

We and others have previously shown that fibronectin deposition on the surface of *S. aureus* allows engagement of α5β1 integrins and triggers the recruitment of actin- and focal adhesion-associated proteins such as paxillin, zyxin, tensin, cortactin, N-WASp, Arp2/3, and FAK to the sites of bacterial attachment [5,15,16]. For several of these proteins, including N-WASP, tensin, FAK and cortactin, a functional role during integrin-mediated uptake of *S. aureus* has been demonstrated [15,16]. As reorganization of the actin cytoskeleton is crucial for the internalization process [9,10,17], it is assumed that dynamic regulation of F-actin by these proteins contributes to bacterial uptake.

Vinculin is one of the characteristic actin-binding proteins recruited to integrin-rich focal adhesion sites, which mechanically links integrin cytoplasmic tails with the actin cytoskeleton [18,19]. Vinculin has no enzymatic activity and its functions are regulated by a conformational switch between a closed (inactive) conformation, mediated by an intramolecular head-tail interaction, and an open (active) state [20]. In the open conformation, the vinculin head and tail domains dissociate, allowing multiple interactions with additional proteins or phospholipids [21]. For example, talin, α-actinin, VASP, paxillin, phosphatidylinositol-4,5-bisphosphate, and F-actin bind to active vinculin [18]. In addition, vinculin may promote actin filament nucleation by recruiting the Arp2/3 complex to integrin tails [22]. Furthermore, depending on the conformational state, vinculin can also act as an F-actin barbed end capping protein [23]. A role for vinculin during bacterial entry has been reported in the case of *Shigella flexneri*. Upon contact with epithelial cells, *S. flexneri* injects the IpaA protein into the host cell cytoplasm, where IpaA directly binds to vinculin inducing a dramatic rearrangement of the actin cytoskeleton to promote bacterial engulfment [24,25]. Vinculin has also been observed to be recruited to *S. aureus*–host cell contact sites or fibronectin-coated beads in epithelial, but not in endothelial cells [15,16,22]. However, it has not been investigated if vinculin has a functional role during integrin-mediated internalization of *S. aureus* into host cells.

In this report, we analyze the contribution of vinculin to FnBP-mediated uptake of *S. aureus* in different human and murine cell types. Surprisingly, re-expression of vinculin in vinculin-deficient fibroblasts as well as shRNA-mediated knock-down of this protein in different cell types do not affect bacterial uptake demonstrating that vinculin is completely dispensable for bacterial internalization via integrin α5β1. Therefore, our results suggest that actin cytoskeleton rearrangements during integrin-mediated endocytosis do not require vinculin function despite the well characterized role of vinculin as a linker between integrins and F-actin.

## Results

### Enhanced cell invasion of *S. aureus* into vinculin knock-out fibroblasts correlates with increased integrin α5 surface expression

**Figure 1:**
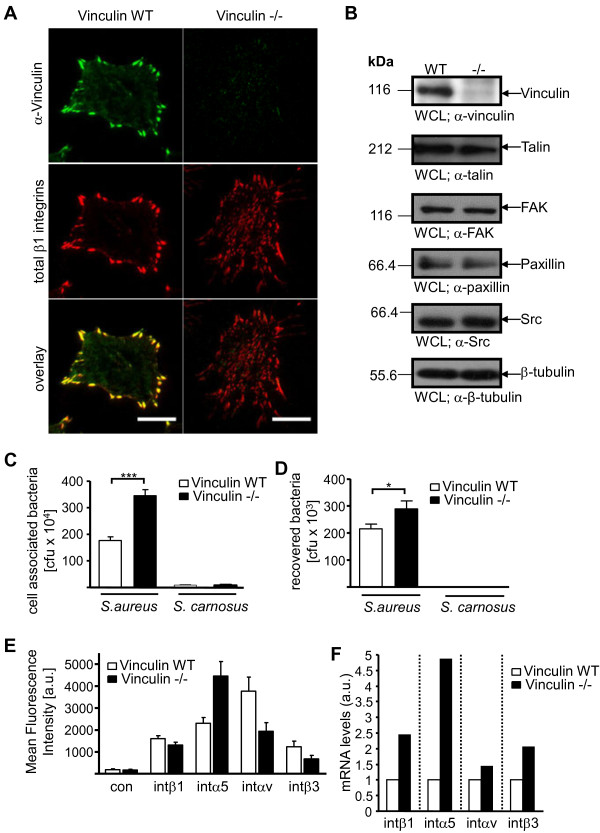
**Increased integrin α5 expression correlates with enhanced bacterial adherence and uptake in vinculin -/- cells. (A)** Vinculin WT and vinculin -/- cells were seeded on fibronectin coated glass bottom imaging dishes, next day fixed and stained for vinculin expression using α-human vinculin antibody (clone hVIN1). Total amounts of integrins were stained using integrin β1 antibody (clone HMβ1-1). TIRF microscopy was used to assess the localization of vinculin and β1 integrins. Bars represent 10 μm. **(B)** Equal amounts of protein in the whole cell lysates (WCL) of vinculin WT and vinculin -/- cells were separated by SDS-PAGE and processed for Western blotting using antibodies against talin, FAK, vinculin, paxillin, Src, or β-tubulin as indicated. Representative blots are shown. **(C and D)** Cells were seeded on gelatine coated 24 well plates and infected with *S. aureus* or *S. carnosus* with MOI 20 for 2 h. Total cell associated or recovered viable intracellular bacteria were determined by bacterial cell adhesion **(C)** or gentamicin protection assays **(D)**, respectively. Values are means ± SEM from 5 independent experiments done in quadruplicate (n=20). Statistical significance of data was assessed by Mann-Whitney test. **(E)** Cells were stained for surface expression of β1, α5, αv, or β3 integrins and examined by flow cytometry. Data are shown as mean fluorescence intensity (MFI) and are corrected for the background MFI of integrin β1 -/- fibroblasts (β1 integrin staining) or corrected for the MFI of cells stained in the absence of primary antibody (α5, αv, and β3 integrin stainings). Results are shown as mean ± SEM from 3 independent experiments. **(F)** mRNA levels of β1, α5, αv, or β3 integrin transcripts in vinculin WT or vinculin -/- cells were analyzed by qRT-PCR. Integrin expression was normalized to GAPDH expression and was set to 1 for vinculin WT cells. Bars represent the mean of two independent experiments each performed in triplicate.

Murine embryonic fibroblasts with a genetic deletion of vinculin (vinculin -/- cells) have been generated and been used in a previous study [26]. When grown on a fibronectin-coated surface, vinculin -/- cells spread more slowly and form smaller, less stable focal adhesions compared to wildtype fibroblasts (vinculin WT cells) or vinculin -/- cells re-expressing vinculin [27,28]. Total internal reflection fluorescence (TIRF) microscopy demonstrates that vinculin is located in well-organized, peripheral focal adhesions in vinculin WT cells. In vinculin -/- cells, no staining with the monoclonal α-vinculin antibody was observed (Figure 1A). The absence of vinculin was accompanied by a reduced size and an altered distribution of integrin β1-containing focal adhesions at the cell-substrate interface (Figure 1A). Compared to vinculin -/- cells, vinculin WT cells showed concentration of large integrin β1-containing focal adhesions in the cell periphery (Figure 1A). However, the overall amount of active integrin β1 did not differ between vinculin WT and vinculin -/- cells as detected by a conformation-specific monoclonal antibody (Additional file 1: Figure S1). The lack of vinculin in whole cell lysates (WCLs) prepared from vinculin -/- cells was also confirmed by Western blotting (Figure 1B). Importantly, expression of several known focal adhesion proteins and vinculin binding partners such as talin, FAK, paxillin, and Src was not altered by the absence of vinculin (Figure 1B). Equal loading of cell lysates for Western blotting was confirmed by probing for β-tubulin (Figure 1B). To test for a functional role of vinculin in the integrin-mediated internalization of bacteria, we infected these fibroblasts with pathogenic *S. aureus* strain Cowan, which connects to the host cell receptor integrin α5β1 by binding to the integrin ligand fibronectin. As a negative control, we used non-pathogenic *S. carnosus*, which lacks a fibronectin binding adhesin and which does not invade host cells [5,15]. As expected, host cell adhesion and invasion assays confirmed that *S. carnosus* was unable to invade fibroblasts, whereas *S. aureus* attached to and invaded vinculin WT cells (Figure 1C and D). Unexpectedly, vinculin -/- cells showed significantly increased numbers of cell-associated *S. aureus* (Figure 1C) and about 40% increase in host cell invasion (Figure 1D). As vinculin -/- cells have undergone several rounds of selection during their establishment [28], compensatory mechanisms might have occurred in the absence of vinculin. In particular, vinculin -/- cells supported increased cell-association of *S. aureus* (Figure 1C), suggesting that the availability of the involved host cell receptor, integrin α5β1, might be altered in these cells. Therefore, expression of different integrin subunits on the surface of vinculin WT and vinculin -/- cells were investigated by flow cytometry. Whereas integrin β1 was present in similar amounts in both cell lines, a strongly increased surface expression of integrin α5 and a reduced expression of the integrin αv subunit was detected in vinculin -/- cells (Figure 1E). Elevated amounts of integrin α5 transcripts were also detected in vinculin -/- cells by quantitative real-time PCR (Figure 1F). The altered integrin α5 expression in vinculin -/- cells correlated with the enhanced host cell binding of *S. aureus*. Together, these results suggested that the altered integrin expression profile of vinculin WT compared to vinculin -/- cells, and not the absence of vinculin, might be responsible for the observed differences in host cell invasion by *S. aureus* in these murine fibroblast lines.

### Re-expression of vinculin in vinculin -/- fibroblasts does not affect bacterial entry

**Figure 2:**
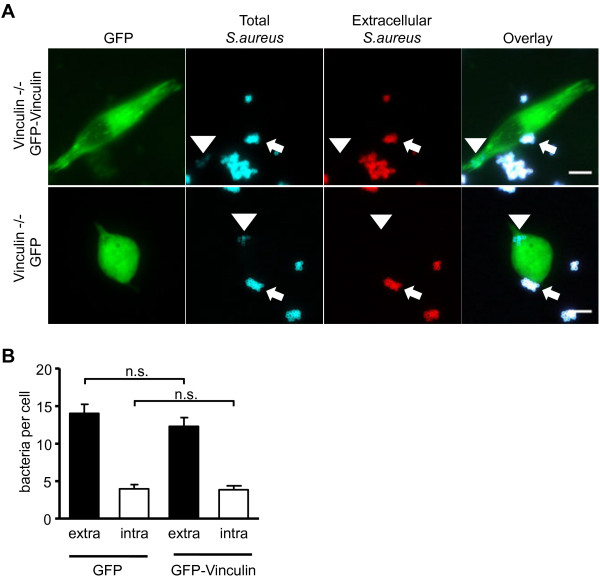
***S. aureus *internalization is not affected by transient expression of vinculin in vinculin -/- cells. (A)** Vinculin -/- fibroblasts were transfected with GFP or GFP-vinculin encoding constructs. 24 h post-transfection, cells were infected with *S. aureus* at MOI 50 for 2 h, fixed and stained for extracellular vs. intracellular bacteria. Representative fluorescence microscopy pictures show intracellular (arrowhead) as well as cell-associated, extracellular (small arrow) *S. aureus*. **(B)** Cells were transfected, infected, and stained as in **(A)** and extra- and intracellular bacteria were quantified. Values are presented as mean ± SEM from 2 independent experiments and based on 100 cells/sample. Data were analyzed for statistical significance using Kruskal-Wallis test.

To address the contribution of vinculin for host cell invasion of *S. aureus* in an identical cellular context, we transiently transfected vinculin -/- cells with a construct encoding enhanced green fluorescent protein (GFP)-tagged murine vinculin. As a control, an GFP encoding vector was used. 48 h post-transfection, cells seeded on gelatine-coated coverslips were infected with *S. aureus* (MOI 50) for 2 h, fixed and, after blocking with 10% fetal calf serum in PBS, extracellular bacteria were stained by rabbit α-staphylococcal serum and goat α-rabbit antibody coupled to Cy5. After cell permeabilization, total cell-associated bacteria were stained by applying rabbit α-staphylococcal serum and Cy3-coupled goat α-rabbit antibody. The double cycle antibody staining according to this established protocol enables the discrimination between intracellular and extracellular bacteria [29,30]. Fluorescence microscopy pictures revealed that a fraction of GFP-vinculin, but not GFP, localized at focal adhesion sites in the transiently transfected vinculin -/- cells (Figure 2A). Moreover, vinculin re-expression was accompanied by a morphological change to a more spread phenotype (Figure 2A). Nevertheless, both GFP as well as GFP-vinculin expressing cells harbored intracellular bacteria (Figure 2A). Enumeration of cell-associated extracellular or intracellular bacteria in GFP versus GFP-vinculin transfected cells demonstrated that bacterial binding and uptake into these cells occurred at a similar level (Figure 2B). Thus, the transient re-expression of vinculin in vinculin -/- cells changes the spreading of the cells, without altering bacterial uptake, indicating that vinculin may not contribute to the internalization of *S. aureus*.

### Vinculin overexpression does not influence the uptake of *S. aureus* in human cells

**Figure 3:**
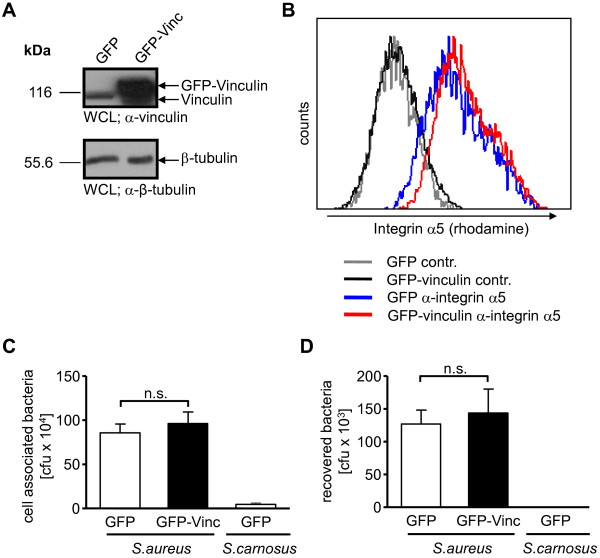
**Transient vinculin overexpression in 293 cells does not interfere with bacterial uptake. (A)** 293 cells were transfected with constructs encoding GFP or GFP-vinculin. 48 h post-transfection whole cell lysates (WCLs) were prepared and analyzed for vinculin and β-tubulin expression by immunoblotting. Representative blots are shown. **(B)** Cells transfected as in **(A)** were compared for integrin α5 surface expression by flow cytometry. Shown is a representative histogram of cells stained with α-integrin α5 antibody and a rhodamine-coupled secondary antibody (blue – GFP; red – GFP-vinculin) or cells stained with the secondary antibody only (grey lines). **(C and D)** Cells as in **(A)** were infected with *S. aureus* and *S. carnosus* for 2 h (MOI 20). Total cell associated or recovered viable intracellular bacteria were determined by bacterial cell adhesion **(C)** or gentamicin protection assays **(D)**, respectively. Values are means ± SEM from 2 independent experiments done in quadruplicate, n=8. Mann-Whitney test was applied for statistical significance testing.

Our results that vinculin might not be necessary for integrin-mediated uptake by murine cells were surprising, given by the fact that vinculin is recruited to the sites of bacterial entry in human cells and has a role in F-actin organization and focal adhesion turnover [15,28]. To analyze weather vinculin is functionally involved in *S. aureus* invasion into human cells, we transiently transfected 293T cells to express GFP or GFP-vinculin, respectively. Western blotting with vinculin antibodies confirmed that total vinculin levels were augmented ~10-fold following transfection with GFP-vinculin (~145 kDa) compared to the endogenous levels of vinculin (~120 kDa) in the GFP transfected cells (Figure 3A). Equal loading of proteins from both samples was confirmed by probing for β-tubulin (Figure 3A). Expression levels of integrin α5 were only slightly increased in response to vinculin overexpression (Figure 3B). Transfected cells were also seeded on gelatine-coated wells and infected at MOI 20 for 2 h with the FnBP-expressing *S. aureus* or the non-invasive *S. carnosus*. Total cell-associated bacteria as well as the numbers of viable intracellular bacteria were then determined. In agreement with our previous observations, the amounts of total cell-associated (Figure 3C), as well as the viable intracellular bacteria were unaltered by vinculin overexpression (Figure 3D). These results demonstrate that vinculin overexpression does not modulate *S. aureus* entry into human cells. These data also indicate that vinculin is not a limiting factor for the integrin-mediated uptake of these fibronectin-binding bacteria and further suggest that the differences observed between murine vinculin WT and vinculin -/- cells might be due to alterations in the expression pattern of integrin subunits.

### ShRNA-mediated vinculin silencing does not interfere with *S. aureus* internalization

**Figure 4:**
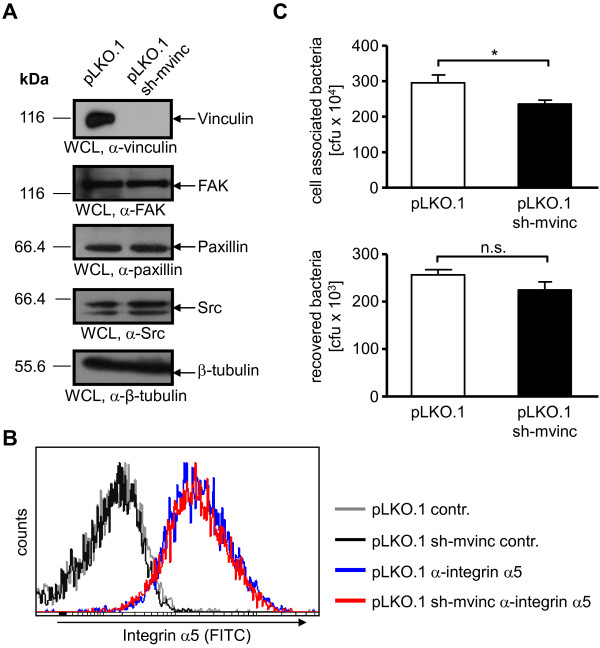
**Knock-down of vinculin in wildtype fibroblasts does not affect*S. aureus *uptake. (A)** Murine fibroblasts were transduced with lentiviral particles harboring either pLKO.1 or pLKO.1 encoding shRNA against mouse vinculin (pLKO-1 sh-mvinc). WCL of the stably transduced cell lines were analyzed for FAK, vinculin, paxillin, Src and β-tubulin protein expression by Western blotting. Representative blots are shown. **(B)** Integrin α5 surface expression in cells from **(A)** was analyzed by flow cytometry. Shown is a representative histogram of cells stained with α-integrin α5 antibodies and a rhodamine-coupled secondary antibody (blue – pLKO.1; red – pLKO.1 sh-mvinc) or cells stained with the secondary antibody only (contr.; grey lines). **(C)** pLKO.1 or pLKO.1 sh-mvinc cells were seeded on gelatine coated 24 well plates. Next day, cells were infected with *S. aureus* at MOI 20 for 2 h. Total cell associated or recovered viable intracellular bacteria were determined by bacterial cell adhesion (top) or gentamicin protection assays (bottom), respectively. Values are means ± SEM from 2 independent experiments done in quadruplicate, n=8. Mann-Whitney test was applied for statistical testing.

Though vinculin is not limiting for *S. aureus* uptake, endogenous levels of this protein might still be needed to support internalization. Therefore, we investigated whether shRNA-mediated knock-down of vinculin might interfere with *S. aureus* uptake. To this end, we generated murine fibroblasts stably transduced with recombinant lentiviral particles harboring the empty plasmid pLKO.1 or the plasmid pLKO.1 encoding shRNA against murine vinculin (pLKO.1 sh-mvinc). After puromycin selection, the successful vinculin knock-down in the transduced cell population was verified by Western blotting. Importantly, complete inhibition of mouse vinculin expression, but unaltered expression of FAK, Src or paxillin was detected (Figure 4A). Furthermore, surface levels of integrin α5 were similar in vinculin-deficient and vinculin-expressing fibroblasts (Figure 4B). Next, we were interested if the vinculin knock-down could affect interaction of the bacteria with host cells with regard to cell attachment and internalization. Accordingly, pLKO.1 and pLKO.1 sh-mvinc fibroblasts were infected with *S. aureus* and the amount of total cell-associated as well as the number of viable intracellular bacteria was determined (Figure 4C). Though the amounts of cell-associated bacteria were decreased in pLKO.1 sh-mvinc fibroblasts compared to control cells, the number of intracellular bacteria in both cell populations was equal. The results from the bacterial adhesion assay correlated with the slightly decreased expression of integrin α5 on the surface of vinculin knock-down cells, compared to control cells (Figure 4B).

**Figure 5:**
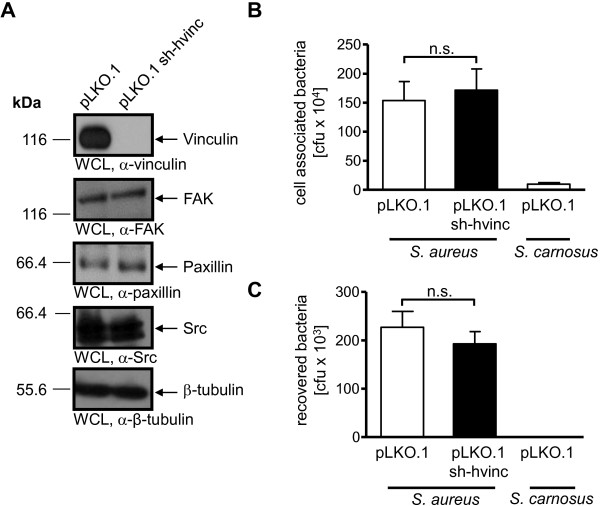
**Vinculin knock-down in 293 cells does not affect the uptake of*S. aureus. *(A)** 293 cells were stably transduced with pLKO.1 or pLKO.1 encoding shRNA against human vinculin (pLKO.1 sh-hvinc). Whole cell lysates were prepared and WCLs were analyzed by Western blotting for vinculin, FAK, paxillin, Src, and β-tubulin expression. Representative blots are shown from 2 independent experiments. **(B and C)** Cells from **(A)** were seeded on gelatine coated 24 well plates and infected with *S. aureus* at MOI 20 for 2 h. Total cell associated or recovered viable intracellular bacteria were determined by bacterial cell adhesion **(B)** or gentamicin protection assays **(C)**, respectively. Values are means ± SEM from 4 independent experiments done in triplicate, n=12. Data were analyzed for statistical significance using Mann-Whitney test.

In a second parallel approach, we generated 293 cells stably transduced with recombinant lentiviral particles harboring the empty plasmid pLKO.1 or with particles harboring plasmid pLKO.1 encoding shRNA against human vinculin (pLKO.1 sh-hvinc). After puromycin selection, pLKO.1 sh-hvinc cells showed no detectable vinculin expression in whole cell lysates, but no alteration in FAK, Src and paxillin expression (Figure 5A). Functional assays of bacterial interaction with host cells revealed that knock-down of vinculin in these human cells again did not influence their ability to support fibronectin-mediated cell attachment or internalization of *S. aureus* (Figure 5B and C).

Taken together, our results demonstrate that vinculin is not required for *S. aureus* invasion in different cell types. Although vinculin might be recruited to the site of bacterial attachment, it has no functional role in orchestrating the integrin-mediated internalization of staphylococci into the eukaryotic host cells.

## Discussion

Since its discovery 32 years ago, vinculin has emerged as a key structural adaptor molecule dynamically linking integrins with the actin cytoskeleton [31]. Here we present experimental evidence that vinculin, despite its reported recruitment, is not required for the integrin-mediated internalization of *Staphylococcus aureus* by mammalian cells. Our conclusions are based on genetic deletion of vinculin and the complementation of the resulting vinculin-deficient cells, shRNA-mediated knock-down of vinculin, and overexpression of vinculin in different cell types, as all these different treatments do not affect host cell invasion by fibronectin-binding *S. aureus*.

Initially, we were puzzled by the finding that vinculin-deficient murine fibroblasts showed slightly elevated association with and uptake of fibronectin-binding *S. aureus*. However, the immortalized vinculin-deficient embryonic fibroblasts exhibited increased expression of integrin α5 compared to wildtype fibroblasts. It is currently unclear, if the altered expression of integrin α5 is directly caused by the lack of vinculin, which has been reported to associate with factors involved in mRNA splicing [32], or if this alteration might be due to prolonged in vitro selection and culture of the vinculin-deficient cells. Clearly, re-expression of vinculin in the vinculin-deficient cells did not alter integrin α5 expression and shRNA-mediated knock-down of vinculin in different cell types did not lead to an increase in integrin α5. Therefore, diminishing or increasing vinculin levels in different cell types did neither affect integrin α5 nor integrin-mediated uptake of *S. aureus* suggesting that changes observed in the vinculin -/- cells might have a secondary cause.

Interestingly, vinculin is involved in cell entry of several bacterial pathogens including *Shigella flexneri* and *Bartonella henselae*. For example, siRNA screening has revealed that vinculin and other integrin-associated molecules are critical for the formation of the characteristic invasome structure, which mediates entry of the zoonotic pathogen *B. henselae* (Bh) into endothelial cells [33].

In the case of *S. flexneri*, a leading cause of dysentery worldwide, the entry into human epithelial cells is dramatically impaired in the absence of vinculin [25]. Upon encounter of host cells, *S. flexneri* injects the IpaA protein. This effector protein contains three vinculin binding sites (VBS), which recognize the N-terminal vinculin head domain [34,35]. Therefore, a single IpaA protein may bind up to three vinculin molecules. Importantly, deletion of IpaA or disruption of all three VBS in IpaA severely impair *Shigella* uptake demonstrating the critical role of vinculin in this entry pathway [35]. Interestingly, the VBS of IpaA are reminiscent of the vinculin binding sites found in the carboxy-terminal rod domain of the focal adhesion protein talin [36]. The eleven VBS of talin also engage the vinculin head domain and help to relief the intramolecular interactions in vinculin [18]. Accordingly, talin or IpaA binding to the vinculin head domain result in exposure of the vinculin tail domain with its binding sites for several proteins including F-actin, α-actinin, or the Arp2/3 complex [35,37]. How vinculin in its active conformation promotes *Shigella* entry is not completely understood, but it has been shown that a vinculin-binding IpaA peptide can modulate the barbed end capping of actin filaments and thereby controls the rate of F-actin polymerization [38]. Similarly, conformational changes in vinculin induced by IpaA may also expose the binding site for the Arp2/3 complex, which is located in a linker region between the vinculin head and tail domain [22]. Again, this would allow *Shigella* to trigger actin polymerization required for efficient bacterial internalization.

A further hint, that vinculin can regulate actin polymerization during bacterial entry comes from the study of *Helicobacter pylori*, an extracellular pathogen, which can engage integrins without being internalized [39]. *H. pylori* translocates the type IV secretion system effector protein CagA into gastric epithelial cells [40]. Bacterial CagA is initially tyrosine phosphorylated and subsequently inhibits the catalytic activity of Src family protein tyrosine kinases [41,42]. The lack of Src activity results in severely reduced phosphorylation of vinculin on residues Y100 and Y1065, disrupting interactions between vinculin and the Arp2/3 complex, and blocking actin cytoskeleton rearrangements in epithelial cells [43]. Together these results suggest that vinculin is a central host factor targeted by several bacterial effector proteins to orchestrate actin cytoskeleton rearrangements to the advantage of the pathogens.

As actin cytoskeleton dynamics are also essential for *S. aureus* host cell entry, yet vinculin does not seem to participate functionally in this process, regulation of actin dynamics might be achieved in a different manner during uptake of fibronectin-binding staphylococci. Previous results indicate that fibronectin-dependent, integrin-mediated internalization of *S. aureus* into host cells requires the integrin-associated tyrosine kinases Src and FAK [10,15,17]. One downstream substrate of the active FAK/Src complex is cortactin [44,45]. Indeed, cortactin is strongly tyrosine phosphorylated upon contact of fibronectin-binding *S. aureus* with host cells and depletion of cortactin reduces *S. aureus* invasion [15]. Recent studies have demonstrated cortactin directly associates with FAK via SH3 domain-mediated binding to proline-rich sequences in FAK [45,46]. As cortactin is also able to organize the F-actin cytoskeleton and to stimulate the actin polymerizing Arp2/3 complex [47,48], recruitment and tyrosine phosphorylation of cortactin might direct the cytoskeletal rearrangements during *S. aureus* internalization, superseding vinculin function downstream of integrin engagement.

Taken together, our investigations come to the surprising conclusion that vinculin is not required for the integrin-mediated uptake of *S. aureus* into host cells. Despite the documented role of vinculin in dynamically linking integrins with the actin cytoskeleton, our results suggest that other cellular factors, such as the FAK-Src-cortactin signaling axis, are critical for triggering actin remodeling and bacterial entry during integrin endocytosis. It also appears likely that uptake of other pathogens, which engage integrins for host cell invasion, might be also independent of vinculin function.

## Methods

### Cell culture and transfection

Human embryonic kidney 293T cell line (293 cells, ACC-635, German Collection of Microorganisms and Cell Cultures, DSMZ, Braunschweig, Germany) was grown in DMEM high glucose (PAA, Pasching, Austria) supplemented with 10% calf serum (CS). Mouse embryonic fibroblasts were grown on gelatine-coated cell culture dishes in DMEM high glucose supplemented with 10% fetal calf serum (FCS; Biochrom, Berlin, Germany), non-essential amino acids and sodium pyruvate. Vinculin wildtype and vinculin -/- cells were generated from mice kindly provided by E.D. Adamson (Burnham Institute, La Jolla, CA) [49]. The fibroblasts were newly derived from mouse embryos and immortalized with SV40 largeT antigen as described [26]. All cell cultures were incubated at 37°C/5% CO_2_ and subcultured every 2 to 3 days. Cells were used between passage 2 and 30, counted and assayed for viability with a Casy Cell Counter (Innovatis, Bielefeld, Germany).

Transfection of 293 cells with expression vectors for GFP (Clontech) or GFP-mouse vinculin [50] was accomplished by standard calcium phosphate co-precipitation using 3.5 μg of plasmid DNA for each 10 cm culture dish as previously described [51]. Fibroblasts were transfected using Lipofectamine 2000 reagent (Invitrogen, Carlsbad, CA) according to the manufacturer’s instructions. Cells were employed in infection experiments 24 to 48 h after transfection.

### Bacteria

*Staphylococcus aureus* Cowan and non-pathogenic *S. carnosus* TM300 have been described before [15]. Bacteria were cultured at 37°C and grown on Tryptic Soybean Broth medium (TSB; BD Biosciences, Heidelberg, Germany). Prior to the experiment bacteria were grown to reach a mid-logarithmic growth phase, washed once with PBS and used for infection at multiplicity of infection (MOI) 20 for gentamicin protection assays and MOI 50 for microscopic evaluation of extra- and intracellular bacteria.

### shRNA construction and lentiviral production

Recombinant lentiviral particles were generated using the plasmids pLKO.1, pMD2.G, and psPAX2 provided by Addgene (www.addgene.org) and maintained in *E. coli* STBL4 (Invitrogen, Carlsbad, CA). Using the algorithm AAGN_18_TT (available online at http://jura.wi.mit.edu/bioc/siRNAext/) sequences that could silence expression of human or mouse vinculin were identified. According to these predictions, complementary primers were synthesized targeting human vinculin mRNA: hVinculin-shRNA-sense 5^′^-ccggaaTCAAGCTGCTTATGAACATctcgagATGTTCATAAGCAGCTTGAtttttttg-3^′^ and hVinculin-shRNA-anti 5^′^-aattcaaaaaaaTCAAGCTGCTTATGAACATctcgagATGTTCATAAGCAGCTTGAtt-3^′^; or targeting murine vinculin mRNA: mVinculin-shRNA-sense 5^′^-ccggaaATCTGGCTGGTACATACACctcgagGTGTATGTACCAGCCAGATtttttttg-3^′^ and mVinculin-shRNA-anti 5^′^-aattcaaaaaaaATCTGGCTGGTACATACACctcgagGTGTATGTACCAGCCAGATtt-3^′^. The oligos were annealed and cloned into the AgeI and EcoRI sites of pLKO.1 generating pLKO.1-sh-hvinc and pLKO.1-sh-mvinc constructs, respectively. The correct insertion of the shRNA cassette was verified by sequencing.

For lentiviral production, 2 × 10^6^ 293 cells were transiently transfected with 14 μg of the respective pLKO.1 vector together with 10 μg of packaging plasmid psPAX2 and 7 μg of envelope-coding plasmid pMD2.G. 48 h later, the virus-containing cell culture supernatant was collected, centrifuged at 2000 rpm at 4°C for 7 min and filtered through a 0.45 μm pore-size filter. 6 ml of the cleared viral supernatant was used to transduce 293 cells or fibroblasts in 10 cm culture dishes. After 24 h, puromycin (0.45 μg/ml for 293 cells and 2.5 μg/ml for fibroblasts) was added and the puromycin-resistant stable cell population was used in experiments after 7 days of selection.

### Quantification of surface integrin expression by flow cytometry

Integrin α5 (clone 5H10-27(MFR5)) and integrin αv (clone RMV-7) antibodies were purchased from BD Biosciences. Integrin β1 (clone CBL1333F) was obtained from Cymbus Biotechnology (London, UK) and integrin β3 antibody (clone 2C9.G3) from eBioscience (San Diego, CA). Secondary antibodies (biotin-SP-conjugated goat α-mouse IgG, biotin-SP-conjugated goat α-rat IgG), streptavidin–FITC and streptavidin-rhodamine (TRITC) were purchased from Jackson ImmunoResearch (West Grove, PA). For quantification of surface integrin expression, suspended fibroblasts were incubated in suspension medium (DMEM containing 0.25% BSA) for 40 min at 37°C. Then, 2 × 10^5^ cells were incubated with appropriate primary antibodies (diluted 1:500) in FACS buffer (5% heat-inactivated FCS, 1% sodium azide in PBS) for 1 h at 4°C. After washing, secondary antibodies were applied for 1h at 4°C, and after washing, samples were analyzed by flow cytometry (LSRII, BD Biosciences).

### qRT-PCR evaluation of integrin mRNA levels

Total RNA was isolated from vinculin wild type and vinculin -/- cells cells using RNeasy Mini Kit (Qiagen, Hilden, Germany). Reverse transcription was performed on 1 μg total RNA. Quantitative real-time PCR was conducted with the sensiMixPlus SYBR Kit (Quantace, Germany) with the following cycle conditions: 95°C for 10 min followed by 40 cycles at 95°C for 10 s, 60°C for 20 s, and 72°C for 20 s. Relative expression of αv-, α5-, β1-, β3-integrins was normalized using glyceraldehyde-3-phosphate dehydrogenase (GAPDH) according to the method by Livak and Schmittgen [52]. The primers used were: mouse αv-integrin (forward: 5^′^-TTGGG GACGA CAACC CTCTG ACAC-3^′^; reverse: 5^′^-TGCGG CGGGA TAGAA ACGAT GAG-3^′^); mouse α5-integrin (forward: 5^′^- CACTT GGCTT CAGGG CATTT C-3^′^; reverse: 5^′^- CAACT ACACC CCCAA CTCAC AGG-3^′^); mouse β1-integrin (forward: 5^′^- TCTCA CCAAA GTAGA AAGCA GGGA-3^′^; reverse: 5^′^- ACGAT AGCTT CATTG TTGCC ATTC-3^′^); mouse β3-integrin (forward: 5^′^- GCTTT GGGGC CTTCG TGGAC AA-3^′^; reverse: 5^′^- CATGG GCAAG CAGGC ATTCT TCAT-3^′^); and mouse GAPDH (forward: 5^′^- TGCAC CACCA ACTGCT TAG-3^′^; reverse: 5^′^- GGATG CAGGG ATGAT GTTC-3^′^).

### Gentamicin protection assay

Briefly, 2 × 10^5^ 293 cells or 1 × 10^5^ fibroblasts were seeded on gelatine coated (0.1% in PBS) 24 well plates. Cells were infected with 20 bacteria per cell (MOI 20) for 2 h. To evaluate the number of intracellular bacteria, the medium was replaced with DMEM containing 50 μg/ml gentamicin. After incubation for 1 h at 37°C, intracellular bacteria were released by treatment with 0.5% saponin in PBS for 10 min at 37°C. Samples were diluted in PBS and plated on TSB agar plates for determination of the recovered colony forming units (cfu). Total cell associated bacteria were determined in separate samples by omitting the incubation with gentamicin and washed once (293) or twice (fibroblasts) with PBS before lysis by 0.5% saponin in PBS.

### Fluorescence staining of vinculin and total internal reflection fluorescence (TIRF) microscopy

3 × 10^4^ vinculin WT and vinculin -/- cells were plated on 4 μg/ml fibronectin coated glass bottom imaging dishes. Next day, cells were washed once with PBS^+/+^ (PBS supplemented with Ca^2+^ and Mg^2+^) and fixed with 4% paraformaldehyde for 20 min. For vinculin staining, fixed samples were washed twice and permeabilized for 10 min in Triton X100 solution (0.5% in PBS). After three washing steps unspecific binding sites were saturated by incubating the samples with blocking buffer (10% heat inactivated FCS in PBS^+/+^) for 10 min. Then, samples were incubated with 1:500 diluted monoclonal mouse α-human vinculin antibody (clone hVIN1; Sigma-Aldrich, Steinheim, Germany) in blocking buffer for 30 min. Samples were washed three times, blocked for 10 min and incubated with biotin-SP-conjugated goat α-mouse IgG antibody (1:500) for 30 min in blocking buffer. Samples were washed again and incubated for 30 min with streptavidin–FITC (1:500) in the dark. All fluorescence staining steps were performed at room temperature. In addition, samples were co-stained for integrin β1 (clone HMβ1-1; BioLegend, San Diego, CA) together with rhodamine conjugated goat-α-armenian hamster IgG antibody (Jackson ImmunoResearch) or for active integrin β1 (clone 9EG7, recognizing the active conformation of integrin β1) together with rhodamine conjugated goat-α-rat IgG (Jackson ImmunoResearch). Antibodies were diluted 1:300 and samples were analyzed by Total Internal Reflection Fluorescence (TIRF) microscopy. Images were acquired using a Leica AF6000LX TIRF system (Leica, Mannheim, Germany) equipped with 100x/1.46 NA Oil HCX PL Apo objective and EMCCD camera (CascadeII:512). Images were digitally processed using ImageJ.

### Cell lysis and western blotting

Cell lysis and Western blotting were performed as described [51] with some modifications. Briefly, protein concentration was assessed using Pierce bicinchoninic assay kit (Thermo Fisher Scientific, Waltham, MA). Equal amounts of proteins were loaded on SDS-PAGE gels. Antibodies against FAK (clone 77) and paxillin (clone 177) were from BD Biosciences, against talin (clone H-300) from Santa Cruz (Santa Cruz, CA) and against vinculin (clone hVIN1) was from Sigma-Aldrich. Antibody against β-tubulin (E-7) and Src were purified from hybridoma cell supernatants. Goat anti-mouse IgG coupled to HRP was purchased from Jackson ImmunoResearch.

### Staining of extra- and intracellular bacteria and fluorescence microscopy evaluation

3 × 10^4^ vinculin -/- cells were seeded on gelatine coated acid washed glass cover slips in 24 well plates. Next day, cells were transfected with GFP or GFP-mouse vinculin using Lipofectamine 2000. 24 h after transfection, cell culture medium was exchanged and cells were infected with *Staphylococcus aureus* (MOI 50) for 2 h, washed twice with PBS^+/+^ and fixed with 4% paraformaldehyde in PBS for 20 min at RT, incubated in blocking buffer for 10 min. Extracellular bacteria were stained using rabbit polyclonal α-staphylococcal serum diluted in blocking buffer (45 min at RT). Afterwards, samples were washed three times and incubated with goat α-rabbit IgG-Cy5 (Jackson Immunoresearch) in the dark for 30 min. After three washing steps, cells were permeabilized using 0.5% Triton/PBS for 10 min, washed three times and blocked additionally for 10 min. Then, after incubation with rabbit polyclonal α-staphylococcal serum, samples were washed three times and incubated with goat α-rabbit IgG coupled to Cy3 in the dark for 30 min. Finally, after three washes with PBS, the coverslips were mounted in embedding medium (DaKo, Glostrup, Denmark) on glass slides and sealed with nail polish. Images were acquired with a Leica AF6000LX fluorescence microscope and processed with ImageJ.

### Statistics

Infection and flow cytometry experiments were performed two to five times, and data were presented as mean ± SEM. Differences in adherence and internalization of staphylococci were analyzed by Mann-Whitney test or for more than 2 groups of analysis by Kruskal-Wallis test. In all analyses, *p* value <0.05 was considered statistically significant.

## Conclusions

This study demonstrates that the focal adhesion protein vinculin does not contribute to the integrin-mediated uptake of fibronectin-binding *S. aureus*. This result is unexpected given the critical role of vinculin as a linker between integrins and F-actin.

## Competing interests

The authors declare that they have no competing interests.

## Authors’ contributions

MB and CRH conceived the study and designed the experiments, MB and YS performed the experiments, AB advised and performed microscopic determinations, SW generated the vinculin knock down cells, WHZ provided reagents and advised on the manuscript, MB and CRH wrote the paper. All authors read and approved the final manuscript.

## Supplementary Material

Additional file 1: Figure S1 Similar distribution of active β1 integrins in vinculin -/- and vinculin WT cells. Vinculin WT and vinculin -/- cells were seeded on Fn coated glass bottom imaging dishes, next day fixed and stained for vinculin using mouse α-human vinculin (hVIN1) antibody, combined with biotin-SP-conjugated goat α-mouse IgG and streptavidin-FITC. In addition, integrin β1 in the ligand-bound, active conformation was detected by rat monoclonal integrin β1 antibody (clone 9EG7) together with rhodamine red conjugated goat-α-rat IgG antibody. TIRF microscopy was used to assess the distribution of vinculin and active β1 integrins. Bars represent 10 μm. Click here for file
