# Supplementary material for: Integrin-mediated internalization of Staphylococcus aureus does not require vinculin
Source: BMC Cell Biol. 2013 Jan 7;14:2. doi: 10.1186/1471-2121-14-2 (PMC3562162; doi:10.1186/1471-2121-14-2)
Supplement: Additional file 1: Figure S1 — Similar distribution of active β1 integrins in vinculin -/- and vinculin WT cells. Vinculin WT and vinculin -/- cells were seeded on Fn coated glass bottom imaging dishes, next day fixed and stained for vinculin using mouse α-human vinculin (hVIN1) antibody, combined with biotin-SP-conjugated goat α-mouse IgG and streptavidin-FITC. In addition, integrin β1 in the ligand-bound, active conformation was detected by rat monoclonal integrin β1 antibody (clone 9EG7) together with rhodamine red conjugated goat-α-rat IgG antibody. TIRF microscopy was used to assess the distribution of vinculin and active β1 integrins. Bars represent 10 μm. [file 1471-2121-14-2-S1.ppt]

## Slide 1
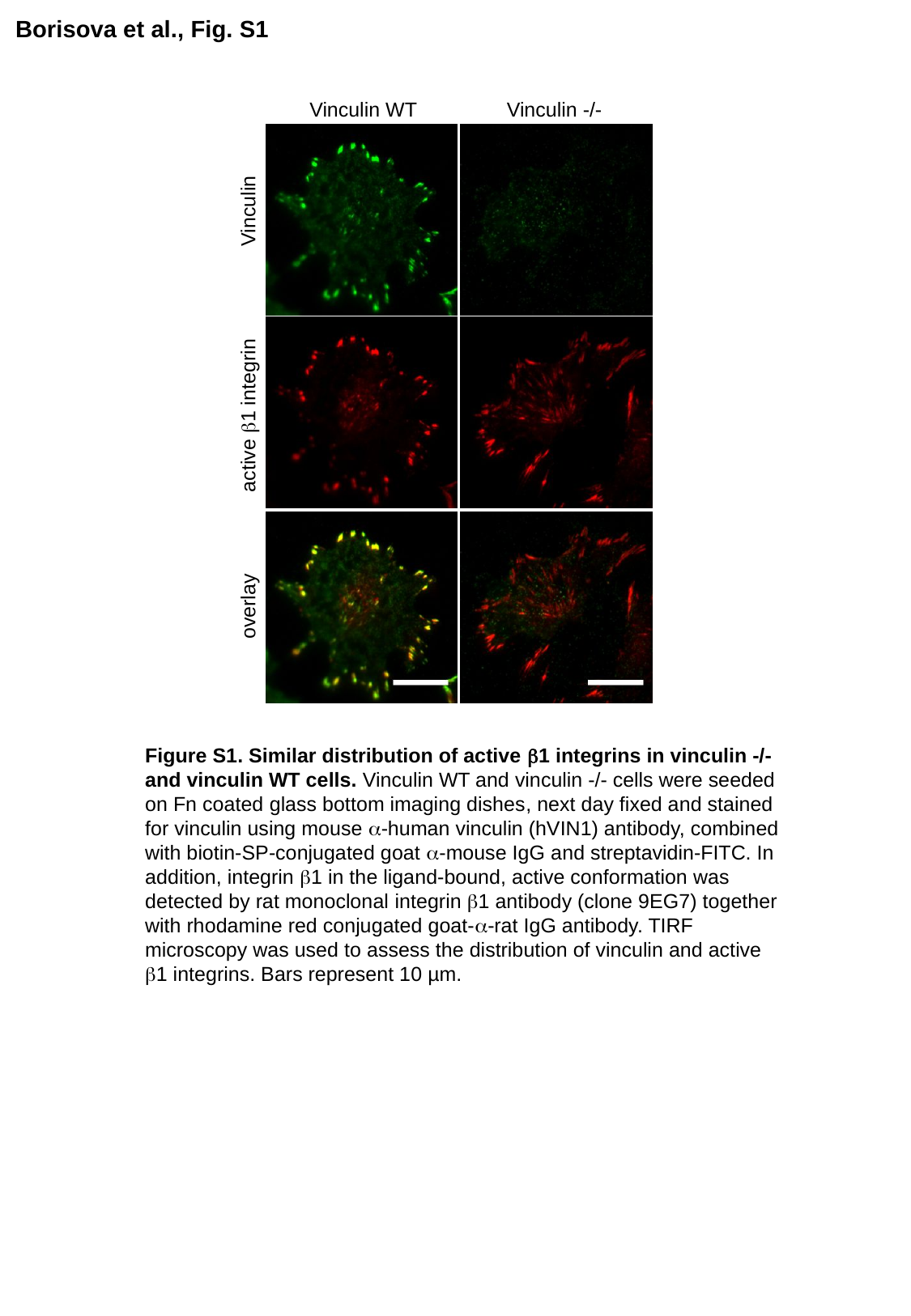

Borisova et al., Fig. S1
Vinculin WT
Vinculin -/-
Vinculin
active β1 integrin
overlay
Figure S1. Similar distribution of active β1 integrins in vinculin -/- and vinculin WT cells. Vinculin WT and vinculin -/- cells were seeded on Fn coated glass bottom imaging dishes, next day fixed and stained for vinculin using mouse α-human vinculin (hVIN1) antibody, combined with biotin-SP-conjugated goat α-mouse IgG and streptavidin-FITC. In addition, integrin β1 in the ligand-bound, active conformation was detected by rat monoclonal integrin β1 antibody (clone 9EG7) together with rhodamine red conjugated goat-α-rat IgG antibody. TIRF microscopy was used to assess the distribution of vinculin and active β1 integrins. Bars represent 10 µm.
